# Investigation of the expression levels of *CPEB4*, *APC*, *TRIP13*, *EIF2S3*, *EIF4A1*, *IFNg*, *PIK3CA* and *CTNNB1* genes in different stage colorectal tumors

**DOI:** 10.3906/sag-2010-18

**Published:** 2021-04-30

**Authors:** Zafer SÖYLEMEZ, Evrim Suna ARIKAN, Mustafa SOLAK, Yüksel ARIKAN, Çiğdem TOKYOL, Hüseyin ŞEKER

**Affiliations:** 1 Department of Medical Biology, Faculty of Medicine, Afyonkarahisar Health Sciences University, Afyonkarahisar Turkey; 2 Department of Medical Genetic, Faculty of Medicine, Afyonkarahisar Health Sciences University, Afyonkarahisar, Turkey; 3 General Surgery Department, Park Hayat Hospital, Afyonkarahisar Turkey; 4 Department of Patology, Faculty of Medicine, Afyonkarahisar Health Sciences University, Afyonkarahisar, Turkey; 5 School of Computing and Digital Technologies, Staffordshire University, Stroke-on-Trent United Kingdom

**Keywords:** Biomarker, colorectal tumor, different stage, gene expression

## Abstract

**Background/aim:**

The aim of the study is to assess expression levels of *CPEB4*, *APC*, *TRIP13*, *EIF2S3*, *EIF4A1*, *IFNg*, *PIK3CA* and *CTNNB1* genes in tumors and peripheral bloods of colorectal cancer patients in stages I–IV.

**Materials and methods:**

The mRNA levels of the genes were determined in tumor tissues and peripheral blood samples of 45 colorectal cancer patients and colon tissues and peripheral blood samples of 5 healthy individuals. Real-time polymerase chain reaction method was used for the analysis.

**Results:**

The mRNA level of the *CPEB4* gene was significantly downregulated in colorectal tumor tissues and was upregulated in the peripheral blood of colorectal cancer patients relative to the controls (P < 0.05). *APC* mRNA level was significantly downregulated in tissues and upregulated in the peripheral blood (P < 0.05). *TRIP13* mRNA level was upregulated in peripheral blood and also significantly upregulated in colorectal tumor tissues (P < 0.05). *EIF2S3* mRNA level was upregulated in tissues and also significantly upregulated in peripheral blood (P < 0.05). *PIK3CA* mRNA level was downregulated in tissues and upregulated in peripheral blood. *EIF4A1* mRNA level was downregulated in tissues and significantly upregulated in peripheral blood (P < 0.05). *CTNNB1* mRNA level was downregulated in tissues and upregulated in peripheral blood. *IFNg* mRNA level was upregulated in both colorectal cancer tumor tissues and peripheral blood.

**Conclusion:**

*TRIP13* and *CPEB4* mRNA up regulation in the peripheral blood of patients with colorectal cancer may be a potential target for early stage diagnosis. In addition to this evaluation, although there is not much study on *EIF2S3* and *EIF4A1* mRNA changes in cases with colorectal cancer, upregulation in peripheral blood draws attention in our study. These data will shed light on the new comprehensive studies.

## 1. Introduction

Cancers originating from the colon or rectum are called colorectal cancer. Colorectal cancer is the third most common cancer type in the world and the fourth most common cause of cancer-related deaths [1]. Early diagnosis is associated with improving prognosis and associated with the identification of genetic biomarkers and the development of available diagnostic tools [2]. The application of gene expression profiling on carcinogenesis studies purposes to identify specific alterations on gene expression according to tumour development and to diagnose and classify tumours on the basis of molecular features [3]. Several studies have been conducted to investigate the difference in gene expression levels between tumor and normal colorectal tissues and have reported significant differences in gene expression profiles between adenoma and normal mucosa. Among these studies, certain groups of genes have been reported to be of differently expressed and consequently help distinguish cancerous tissues from normal ones [4–10]. Ortiz-Zapater et al. [11] reported that *CPEB4*-associated mRNAs are significantly enriched in a number of cellular functions that are relevant to tumorigenesis. The adenomatous polyposis coli (*APC*) gene is a key tumor suppressor gene. Mutations in *APC* gene are the basis of hereditary predisposition to colorectal cancer in familial adenomatous polyposis coli (FAP) and also the primary reason for the formation of sporadic colorectal tumors. Mutant *APC* may also impair cytoskeleton adhesion and stability, which play a role in cancer progression. A better understanding of both genetics and biological function of *APC* may help develop preventive or therapeutic regimes that aim to reduce the burden of colorectal cancer over time [12]. Somatic mutations in the *CTNNB1* gene have been identified in several types of cancer including include colorectal, liver, thyroid, ovarian, endometrial and skin cancers and medulloblastoma. *TRIP13* has been found to play a key role in meiotic recombination, spindle checkpoint and chromosome synapses [13]. Studies have shown that *TRIP13* is overexpressed in multiple neoplasms [14–16]. Sheng et al. [17] suggested that *TRIP13* can support colorectal cancer cell proliferation, migration and invasion in vitro, and reported low survival times for colorectal cancer patients. *IFNg* is a critical proinflammatory cytokine for natural and adaptive immunity against viral and intracellular bacterial infections and tumor control. *IFNg* is also important for the activation of macrophages in response to intracellular bacteria and viruses. Decreased *IFNg* induction or signal has also been demonstrated to be associated with increased sensitivity to intracellular bacteria, some viruses and tumor onset [18]. *PIK3CA* is a proto-oncogene encoding phosphatidylinositol-3-kinases (PI3K) located in the EGFR tyrosine-kinase domain and leads to the activation of AKT’s phosphorylation and the AKT-mTOR signal path. The phosphoinositol-3-kinase (PI3K) pathway has been discovered as an enzymatic activity associated with a viral oncoprotein in human cancers. This pathway has attracted a lot of attention in human cancer studies because it is important for cell cycle, proliferation, growth, survival, protein synthesis, and glucose metabolism [19]. The *EIF2S3* gene encodes the core subunit of eukaryotic translation initiation factor-2 (eIF2), a heterotrimeric GTP binding protein involved in the incorporation of methionyl-tRNA (i) into the 40S ribosomal subunit. EIF2 complex is required for protein synthesis [20]. EIF4A is a member of the DEAD box protein family and functions as an ATP-bound RNA helicase to catalyze the dissolution of the mRNA secondary structure at 5’UTR [21].

Among all the genes reported in the literature with their potential cause in tumor development, *CPEB4*, *APC*, *TRIP13*, *EIF2S3*, *EIF4A1*, *IFNg*, *PIK3CA* and *CTNNB1* have particularly been identified to be a set of potential candidates for tumor development. Therefore, in this study, they have been experimentally studied by considering their expression level in 45 colorectal cancer patients who were at different stages of their disease. It is discovered that these genes exist in tumors and peripheral bloods, but with varying expression levels, which appears to suggest that they may help distinguish patients and their disease stages, consequently understand molecular mechanism of the disease.

## 2. Materials and methods

### 2.1. Sample collection and clinicopathological information

Fifty patients who underwent surgical resection in the Department of General Surgery, Afyonkarahisar Health Sciences University between April 2018 and December 2019 were chosen for the study. Tissues and blood samples were obtained from the patients with histopathologically confirmed colorectal carcinoma (26 male and 19 female patients). Of the fifty patients, five are noncolorectal cancer subjects and selected as controls. The stage of cancer was estimated on the basis of the TNM and American Joint Committee on Cancer classifications.

### 2.2. RNA extraction, real-time PCR and RT-PCR analyses

The fresh samples were transported in liquid nitrogen and stored in –80 °C until RNA extraction. About 5 mL peripheral blood samples were stored in EDTA-coated vacutainers and RNA extractions were immediately performed. RNA extractions of tissues and peripheral blood samples were performed by using EZ-RNA Total RNA extraction kit (BI, Israel, Cat. No: 20-400-100) according to the manufacturer’s protocol. Then, RNA amount and RNA purity were quantified for each RNA sample by Nanodrop ND-1000 spectrophotometer V3.7. RNA samples were stored at –80 °C until use. All the RNA samples were reverse transcribed into cDNA from 1 μg of total RNA (iScript Reverse Transcription Supermix, Bio-Rad Laboratories, Hercules, CA, USA, Cat. No: 170884) under the following conditions: One cycle at 25 °C for 5 min, 46 °C for 20 min and 95 °C for 1 min. Real-time PCR was performed after reverse transcription. mRNA expression analysis of all the genes was performed by using the Rotor Gene-Q (Qiagen, Hilden, Germany). cDNAs that belong to the cases were added to iTaq Universal SYBR Green Supermix (Biorad Laboratories, Kat. No: 1725122) according to the manufacturer’s protocol. Oligonucleotide primers were designed by Oligomere Biotechnology (Ankara, Turkey) based on following primer sequences:

*CPEB4*-F: 5’-CATATTCAGCTCCAGAAGTATGCTC-3’

*CPEB4*-R: 5’-AGTGCATGTCGAATGTCCTG-3’

*APC*-F: 5’-AAAATGTCCCTCCGTTCTTATGG-3’

*APC*-R: 5’-CTGAAGTTGAGCGTAATACCAGT-3’

*TRIP13*-F: 5’-ACTGTTGCACTTCACATTTTCCA-3’

*TRIP13*-R: 5’-TCGAGGAGATGGGATTTGACT-3’

*EIF2S3*-F: 5’-GTATCACTTTTTGCGGAGCAT-3’

*EIF2S3*-R: 5’-GGGGTCAATTTTTGTTCCAA-3’

*EIF4A1* F: 5’-AAGGCGTCATCGAGAGTAACT-3’

*EIF4A1* R: 5’-ATGTGGCCGTTTTCCCAGTC-3’

*IFNg*-F: 5’-TCAGCTCTGCATCGTTTTGG-3’

*IFNg*-R: 5’-GTTCCATTATCCGCTACATCTGAA-3’

*PIK3CA*-F: 5’-CCTGATCTTCCTCGTGCTGCTC-3’

*PIK3CA*-R: 5’-ATGCCAATGGACAGTGTTCCTCTT-3’

*CTNNB1*-F: 5’-CTTGCTCAGGACAAGGAAGC-3’

*CTNNB1*-R: 5’-CATATGTCGCCACACCTTCA-3’

*GAPDH-F*: 5’-CATTGCCCTCAACGACCACTTT-3’

*GAPDH-R*: 5’-GGTGGTCCAGGGGTCTTACTCC-3’.

We used the following RT-PCR protocol for *CPEB4*, *APC*, *TRIP13*, *EIF2S3*, *EIF4A1*, *CTNNB1*: 95 °C for 30 s initial denaturation followed by 40 cycles of 95 °C for 5 s and 60 °C for 30 s, and for *IFNg*, *PIK3CA*: 95 °C for 30 s initial denaturation followed by 40 cycles of 95 °C for 5 s and 63 °C for 30 s. Melting curve analysis was performed for confirmation of single product amplification at the end of the PCR. 65–95 °C, 0.5 °C increments at 5 s/step. Each run has been performed triplicate.

### 2.3. Statistical analysis

All the data analyses were performed using REST 2009 V2.0.13 and SPSS v.19 software which use pairwise fixed reallocation randomization test [22] where P < 0.05 is deemed to represent a statistically significant result. REST 2009 Software is a standalone tool for analysis of gene expression data from quantitative, real-time PCR experiments. The analysis or quantitation of relative gene expression uses expression of reference genes to normalize expression levels of genes of interest in different samples.

## 3. Results

The study included 45 patients (average age: 66.6 ± 12.66) with pathologically proven colorectal carcinoma and 5 control patients (average age: 62.5 ± 11.08). Cancer tissues and blood samples were collected for each of the cases. In colorectal cancer, tumor localization was in the rectum for 17 patients and in the colon for 26 patients. Of 45 patients, the number of patients at stages I, II, III and IV are 8, 17, 15 and 5, respectively.

### 3.1. Gene expression analysis

The mRNA levels of *CPEB4*, *APC*, *TRIP13*, *EIF2S3*, *EIF4A1*, *IFNg*, *PIK3CA* and *CTNNB1* genes expressed in colorectal cancer tissue specimens, colorectal cancer blood samples, normal colon tissues and blood samples were analysed.

#### 3.1.1. mRNA analysis of *CPEB4*, *APC*, *TRIP13*, *EIF2S3*, *EIF4A1*, *IFNg*, *PIK3CA* and *CTNNB1* genes expressed in normal and cancer tissues

Changes in mRNA levels of related genes expressed in tumor tissues of colorectal cancer (CRC) cases were determined according to the tissues of the control group. While the mRNA levels of *CPEB4* and *APC* genes decreased significantly compared to the control group (0.512 and 0.594 fold regulation value, respectively) (P < 0.05), the mRNA level of the *TRIP13* gene significantly increased (2.139) (P < 0.05). The mRNA levels of *EIF2S3* and *IFNg* genes increased compared to the control group, while the mRNA level of *EIF4A1*, *PIK3CA* and *CTNNB1* genes decreased (Figure 1).

#### 3.1.2 mRNA analysis of *CPEB4*, *APC*, *TRIP13*, *EIF2S3*, *EIF4A1*, *IFNg*, *PIK3CA* and *CTNNB1* genes expressed in normal and cancer peripheral blood samples

Changes in mRNA levels of related genes expressed in peripheral blood samples of colorectal cancer cases were determined according to the peripheral blood samples of the control group. The mRNA levels of the *CPEB4*, *APC*, *EIF2S3* and *EIF4A1* genes were significantly increased compared to the control group (2.467; 2.066; 1.852; 1.522 fold regulation value; respectively) (P < 0.05). The mRNA levels of *TRIP13*, *IFNg*, *PIK3CA* and *CTNNB1* genes also increased (Figure 2).

#### 3.1.3. mRNA analysis of *CPEB4*, *APC*, *TRIP13*, *EIF2S3*, *EIF4A1*, *IFNg*, *PIK3CA* and *CTNNB1* genes expressed in tumor tissues of stages I–IV colorectal cancer patients

Changes in mRNA levels of related genes expressed in tumor tissues of stages I–IV colorectal cancer cases were determined according to the tissues of the control group. The findings for each stage are as follows:

**Stage I:** The mRNA levels of the *CPEB4* and *CTNNB1* genes decreased significantly compared to the control group [0.250 (P < 0.001); 0.204 (P < 0.05) fold regulation value; respectively]. While *TRIP13* and *EIF2S3* mRNA levels increased compared to the control group, mRNA levels of *APC*, *EIF4A1*, *IFNg* and *PIK3CA* genes decreased (Figure 3).

**Stage II:** While mRNA levels of *CPEB4*, *APC*, *PIK3CA* and *CTNNB1* genes decreased compared to the control group, *TRIP13*, *EIF2S3*, *EIF4A1* and *IFNg* mRNA levels increased (Figure 4).

**Stage III:** mRNA levels of *CPEB4*, *APC*, *EIF4A1*, *PIK3CA* and *CTNNB1* genes decreased compared to the control group, whereas *TRIP13*, *EIF2S3* and *IFNg* mRNA levels increased (Figure 5).

**Stage IV:** The mRNA level of the *APC* gene significantly decreased compared to the control group [0.333 fold regulation value, (P < 0.05)]. In addition, *CPEB4*, *EIF2S3*, *EIF4A1* *PIK3CA* and *CTNNB1* mRNA levels decreased compared to the control group, while *TRIP13* and *IFNg* mRNA levels increased (Figure 6).

#### mRNA analysis of *CPEB4*, *APC*, *TRIP13*, *EIF2S3*, *EIF4A1*, *IFNg*, *PIK3CA* and *CTNNB1* genes expressed in peripheral blood samples of stages I–IV colorectal cancer patients

Changes in mRNA levels of related genes expressed in peripheral blood samples of stages I–IV colorectal cancer cases were determined according to the peripheral blood samples of the control group. The findings for each stage are as follows:

**Stage I:** The mRNA levels of *CPEB4*, *TRIP13*, *EIF2S3*, *CTNNB1*, *EIF4A1* genes significantly increased compared to the control group [2.803; 3.553; 2.507; 3.548 (P < 0.05); 2.441; (P < 0.001) fold regulation value; respectively]. The mRNA levels of the *APC*, *IFNg* and *PIK3CA* genes also increased compared to the control group (Figure 7).

**Stage II:** The mRNA levels of *CPEB4* and *TRIP13* genes significantly increased compared to the control group [2.788; 1.943 fold regulation value (P < 0.05)]. The mRNA levels of the *APC*, *EIF2S3*, *EIF4A1* and *CTNNB1* genes also increased compared to the control group, while the mRNA levels of the *IFNg* and *PIK3CA* genes decreased (Figure 8).

**Stage III:** The mRNA levels of *APC*, *TRIP13*, *EIF2S3* and *EIF4A1* genes significantly increased compared to the control group [2.47; 2.696; 2.32; 1.838 fold regulation value (P < 0.05)]. The mRNA levels of *CPEB4*, *IFNg*, *PIK3CA* and *CTNNB1* genes also increased compared to the control group (Figure 9).

**Stage IV:** While mRNA levels of *CPEB4*, *APC* and *PIK3CA* genes increased compared to the control group, *TRIP13*, *EIF2S3*, *EIF4A1*, *IFNg* and *CTNNB1* mRNA levels decreased (Figure 10).

## 4. Discussion

In colorectal cancer, a comprehensive list of biomarkers with quite different expression patterns can be used as molecular markers to complement existing histopathological factors in patients’ follow-up and treatment strategies. Although screening tests are becoming increasingly important, colon cancer cases are often diagnosed at an advanced stage of the tumor, where the chances of survival are greatly reduced. It is well known that various gene expression differences can be detected during colon cancer development. On the basis of all genome expression studies, it is intended to identify clinically useful biomarkers and then be developed and used as part of routine diagnosis in tumor classification [23].

### 4.1. Relationship between colorectal cancer and *CPEB4* gene expression

Abnormal expression of *CPEB4* is associated with certain types of cancer, suggesting that *CPEB4* can play critical roles in the control of cancer proliferation and metastasis [24]. In particular, it is suggested that *CPEB4* plays an important role in the migration and invasion of cancer cells in certain types of cancer, and can be used as a target for cancer treatment [11, 24–26]. In addition, it is of great interest to identify cancer-associated RNA-binding proteins, to understand colorectal cancer biology, and to potentially set new goals with cancer treatment and prognostic biomarkers [27]. It has been reported that colorectal cancer tissues express high levels of *CPEB4* and that high mRNA level is associated with advanced tumor stage, lymph node metastasis, distant metastasis and poor prognosis in patients with colorectal cancer [28]. In our study, *CPEB4* mRNA level was significantly decreased in all colorectal tumor tissues. However, similar to our study, Xu and Liu [24] reported that *CPEB4*’s mRNA level decreased compared to control in prostate cancer and adjacent tissues. Considering the studies, there are different results related to *CPEB4* in different types of tumor tissues. *CPEB4* is thought to affect tumor growth, invasion and vascularization by applying preoncogenic effects, since the high level of expression of *CPEB4* has been defined in a wide variety of malignancies [11]. In addition, *CPEB4* gene expression has been reported to be relatively low in NSCLC samples compared to adjacent non-cancerous tissues [29]. In another study, *CPEB4* gene expression was reported to increase in pancreatic ductal carcinoma [11] but decreased in hepatocellular carcinoma [30]. When liver samples taken from 125 hepatocellular carcinoma patients were compared with 49 controls, it was reported that the protein level of *CPEB4* increased in early stage hepatocellular carcinoma and decreased in late stage hepatocellular carcinoma [31]. These changes in the expression of *CPEB4* during the progression of hepatocellular carcinoma suggest that it plays a complex role in tumorigenesis. It has been suggested that *CPEB4* gene expression is directly proportional to the pathological degree of glioma, increased *CPEB4* gene expression in primary tumors in predicting poor outcomes in patients, and suppressed *CPEB4* gene expression inhibits tumor cell proliferation and is a potential therapeutic target for glioblastoma [32]. However, Hu et al. [33] reported that *CPEB4* gene expression increases significantly in glioma and this increase is directly proportional to the advanced cancer stage. It has been reported that for patients with glioma, *CPEB4* may be a highly sensitive prognostic indicator. It is thought that *CPEB4* is over-expressed in a wide variety of tumors, including colorectal cancer, skin cancer and kidney cancer, and high expression of *CPEB4* may also be effective in tumor development. It has been suggested that *CPEB4* is important in tumor invasion and metastasis processes, and high expression level is an indicator for poor outcome in colorectal cancer patients [34]. In addition, Xu and Liu [24] reported a similar result in invasive and metastatic cancers. In addition, *CPEB4* has also been reported to be highly expressed in the peripheral blood of cases with colorectal cancer [35]. Similar to this study, in our study, *CPEB4* mRNA level increased significantly in the peripheral blood of patients with colorectal cancer. When compared in terms of stages, this increase was important in stages I and II. This suggests that *CPEB4* gene expression in peripheral blood from the early stages of colorectal cancer may be an indicator for colorectal cancer. It has been suggested that such gene expression changes may be related to different pathways regulated by *CPEB4* in different types of cells. It has been suggested that such gene expression changes may be related to different pathways regulated by *CPEB4* in different types of cells [29]. In our study, while the mRNA level of the *CPEB4* gene was significantly decreased in all colorectal tumor tissues of the cases, it was observed that it is increased significantly in peripheral blood samples. When evaluated comparatively in terms of stages, the increase in the mRNA level of *CPEB4* gene was found to be statistically significant in the peripheral blood samples of cases in colorectal cancer stages I and II. This suggests that the increase in *CPEB4* mRNA level in peripheral blood since the early stages of colorectal cancer may be a potential biomarker for colorectal cancer.

### 4.2. Relationship between colorectal cancer and *APC* gene expression

Although there are many studies related to *APC* mutations in literature searches, there is not much study on gene expression. Birnbaum et al. [36] investigated the role of the *APC* gene in the 183 colon adenocarcinoma series, by combined analysis of gene expression, mutation, allelic loss, and promoter methylation and metastasis formation. While spot mutations were detected in 73% of cases and allelic losses in 39% of cases; 59% of tumors showed bialelic inactivation. No relationship was found between the number and type of *APC* gene expression changes and metastatic transformation. The results show that determining the *APC* status cannot help for the prediction of metastasis and cannot be used to classify stage II colon cancers. In our study, the mRNA level of the *APC* gene was significantly decreased in all colorectal tumor tissues, while the peripheral blood of the same cases increased significantly. When compared in stages, this increase was also important in stage III. When evaluated in terms of stages, while the increase in the mRNA level of the *APC* gene in the peripheral blood samples of the patients in colorectal cancer stage III, the decrease in stage IV tumor tissues was significant. These results indicate that determining *APC* mRNA levels cannot assist in predicting colorectal cancer and cannot be used to classify the stages of colon cancer.

In the study of Güler [37], 8 of 20 patients with colorectal carcinoma reported that there was a mutation in the *APC* gene, whereas in the rest, the expression of the *APC* gene was significantly different compared to the control group. It has been suggested that *APC* is overexpressed in NIH3T3 fibroblast cells to block cell cycle progression from serum-induced G0/G1 to S phase [38]. Consistent with these data, it has been suggested that *APC* is overexpressed to prevent transition to the G1 phase in colorectal cancer cell lines. This function is partially associated with the regulation of the β-catenin/ Tcf mediated transcription of S-phase regulators such as cyclic D1 and c-myc [39]. It has been suggested that *APC* can also affect proliferation independently of β-catenin. Thus, deactivation of the mutant *APC* at the G1/S control point can contribute to aberrant cell proliferation. Copy number changes, regulatory changes, deletions, severe mutations and other potential causes are difficult to distinguish through *APC* mRNA expression data. In other words, more studies are needed to demonstrate causality correlation with respect to *APC* mRNA changes. The results obtained as a result of mRNA analyses are not sufficient for any necessary information such as mutation status and other clinicopathological features. Further study is therefore required for developing preventive or therapeutic strategies that may be developed over time, especially aimed at reducing the colorectal cancer burden.

### 4.3. Relationship between colorectal cancer and *CTNNB1* gene expression

The *CTNNB1* gene encodes β-catenin. β-catenin plays an important role in the adhesion of cells and communication between cells. Somatic mutations in the *CTNNB1* gene have been identified in many types of cancer. If β-catenin does not phosphorylate and therefore does not break down, it accumulates in the cellular cytoplasm and nucleus. The accumulation of the β-catenin may result from the Wnt signal [40] by inactivation of the *APC* or direct mutation of the β-catenin itself [41]. Mutations in the *APC* or *CTNNB1* genes inhibit GSK3β-mediated phosphorylation followed by β-catenin degradation [42] and result in activation of the catenin transcription [43]. This critical role of Wnt in intestinal homeostasis is the basis for understanding why Wnt path deregulation contributes to colorectal carcinogenesis. Of the known Wnt signal cascades, Wnt/β-catenin (canonical pathway) mutates in about 90% of colorectal cancers. These mutations are mainly found in the genes of *APC* and β-catenin and both lead to pathway activation, but other path components may also harbor mutations [44]. Signal activation of Wnt-β-catenin leads to accumulation of catenin, which can be detected in >80% of colorectal cancer tumors in the nucleus [45]. In addition, high nuclear catenin levels have been correlated with poor prognosis in colorectal cancer patients [46]. In our study, *CTNNB1* mRNA level decreased in tumor tissues compared to the control group, while it increased in peripheral blood samples. In particular, different mRNA levels were found in stage I colorectal cancer tissues and peripheral blood samples. These different results we obtained support the view that the changes in *CTNNB1* mRNA level may not be useful in colorectal cancer diagnosis.

### 4.4. Relationship between colorectal cancer and *TRIP13* gene expression

*TRIP13* has been found to play a key role in meiotic recombination, spindle checkpoint and chromosome synapses [13]. Studies have shown that *TRIP13* is overexpressed in multiple neoplasms [14–16]. *TRIP13* has been shown to be a localized protein in the kinetochore that allows cell division to progress correctly. A number of kinetochore-localized proteins are highly synthesized in various cancers, and their expression is associated with genomic imbalance or malignant transformation of cancer cells [47]. Although it plays an important role in meiotic regulation, excessive expression or amplification of *TRIP13* has been found in more than one human cancer [48,49].

In our study, *TRIP13* mRNA level increased significantly in both colorectal tumor tissues and peripheral blood compared to the control group. Similarly, Kurita et al. [50] analyzed the mRNA level of *TRIP13* between normal and tumor tissues. They suggest that *TRIP13* is involved in colorectal cancer cell proliferation and invasion, and may be a potential indicator for colorectal cancer treatment. Sheng et al. [17] analyzed multiple colorectal cancer datasets available from Oncomine to determine the expression profile of TRC13 in colorectal cancer, and found that gene expression of *TRIP13* increased in tumor tissue compared to that of normal tissue. To confirm the results, 41 pairs of colorectal cancers and TCGA (Cancer Genome Atlas) examined the mRNA level of *TRIP13* in the corresponding normal tissues, and reported that *TRIP13* was expressed in tumor tissue at a high rate (P < 0.001).

In our study, the increase in *TRIP13* mRNA level is important especially in peripheral blood stages I, II, and III. In the development of colorectal cancer, high mRNA level of *TRIP13* can be observed from the early stage. Sheng et al. [17] reported that high *TRIP13* expression was significantly associated with advanced pTNM stage. High *TRIP13* expression has been shown to reveal poor course in other carcinomas such as renal renal clear cell carcinoma, renal papillary cell carcinoma, brain low grade glioma, liver hepatocellular carcinoma in total survival (OS: overall survival) analysis. Therefore, abnormal expression of *TRIP13* is a common occurrence in cancer cells. It shows a potential oncogenic role of *TRIP13* in cancer development [51]. Considering the findings mentioned above, *TRIP13* appears to contribute to tumor formation and tumor progression in various human cancers. In human mycosis fungoides tumor, *TRIP13* gene expression increased compared to control biopsies [52].

What is important here is that *TRIP13* gene expression and activity are required for accurate chromosome segregation. It is strongly suggested that *TRIP13* is an oncogene when it is possible to monitor the suitability of chromosome segregation with various pathways and its effects on cell physiology [50]. Our results support this view. In our study, *TRIP13* mRNA levels increased significantly in both colorectal tumor tissues and peripheral blood samples compared to controls. This increase in peripheral blood samples, especially in cases of colorectal cancer stages I, II, and III, seems to be significant. An increase in the level of mRNA of *TRIP13* can be observed in the development of colorectal cancer from an early stage. *TRIP13* strongly suggests that overexpression may be a common phenotype in colorectal cancer and a potential finding/biomarker for early stage colorectal cancer diagnosis.

### 4.5. Relationship between colorectal cancer and *IFNg* gene expression

Interferons can also have a bidirectional effect on cancer cell behavior, such as promoting proliferation or growth inhibition. Indeed, contradictory results have been reported regarding the interferons function as tumor promoters or tumor suppressors in melanoma and colorectal cancer. The differences may arise from different experimental environments, such as the effect of the microenvironment, the amount and quality of the immune infiltrate, and the mutation status of cancer cells. Therefore, there is a need to better understand the biology of interferons in cancer and analyze the data depending on the conditions [53]. In our study, *IFNg* mRNA levels increased in tumor tissues and peripheral blood samples of colorectal cancer group compared to the control group. However, this increase was not statistically significant. When the data were evaluated in terms of stages, different changes were observed in the mRNA levels according to the data of the control group individuals. Studies to clarify the effect of *IFNg* on the colorectal cancer process are very new and future studies are needed.

### 4.6. Relationship between colorectal cancer and *PIK3CA* gene expression

*PIK3CA* is a proto-oncogene encoding phosphatidylinositol-3-kinases (PI3K) located in the EGFR tyrosine-kinase domain. It leads to phosphorylation of AKT (protein kinase B) and activation of the AKTmTOR signaling pathway. The phosphoinositol-3-kinase (PI3K) pathway has been discovered as an enzymatic activity associated with a viral oncoprotein in human cancers. This pathway has attracted a lot of attention in human cancer studies because it is important for cell cycle, proliferation, growth, survival, protein synthesis and glucose metabolism [19]. In our study, it was found that *PIK3CA* mRNA levels decreased in colorectal tumor tissues compared to control, while it increased in peripheral blood compared to the control group. However, these changes are not statistically significant. *PIK3CA*, the catalytic subunit of PI3K, undergoes mutation in many different tumors, including colorectal cancer [54,55]. *PIK3CA* mutations have been reported in about 80% of mutations in 10%–20% of colorectal cancers, exon 9 and exon 20 at two hot spots [55]. It has been suggested that *PIK3CA* mutations may be a long-sought biomarker for successful adjuvant therapy with aspirin in colorectal cancer patients. Therefore, *PIK3CA* mutations appear to be a promising biomarker; however, they reported that more studies are needed to precisely define the effect of somatic mutations in the *PIK3CA* gene in the treatment of colorectal cancer patients [56].

Yan et al. [57] investigated the potential value and mechanism of *PIK3CA* mutation in colorectal cancer chemotherapy. First line chemotherapy response and *PIK3CA* mutation correlation were evaluated and evaluated in 440 colorectal cancer patients in medical records. The frequency of *PIK3CA* gene mutation in colorectal cancer patients has been found to be 9.55%, and this has been reported to be associated with late TNM staging and low histological grade. Colorectal cancer patients with the *PIK3CA* mutation have been reported to respond poorly to primary chemotherapy than those without the *PIK3CA* mutation. *PIK3CA* mutation tumor cells showed poor sensitivity to first-line chemotherapy in vitro and in vivo. The findings showed that PI3K/Akt activation induced by the *PIK3CA* mutation contributes to the survival and proliferation of colorectal cancer stem cells, in which cells are more resistant to chemotherapy. In colorectal cancer studies, conflicting results have been reported about the use of *PIK3CA*, which may be a predictive marker for treatment. Recent metaanalyses have shown that mutations in *PIK3CA* exon 20 may be a marker for resistance to anti-EGFR treatment [58,59].

When the outcomes of the studies are evaluated, mutation analyses come to the fore rather than *PIK3CA* mRNA expression analysis. However, the relationship between *PIK3CA* mutations and the prognosis of colorectal cancer patients remains unclear.

In our study, *PIK3CA* mRNA levels decreased in colorectal tumor tissues compared to control, while in peripheral blood samples increased compared to the control group. However, these changes were not statistically significant. When the outcomes of the studies are evaluated, in *PIK3CA*; mutational analysis is more prominent than mRNA analysis. However, the relationship between *PIK3CA* mutations and the prognosis of colorectal cancer patients remains controversial. In early diagnosis of patients with colorectal cancer, mRNA analyses associated with mutation analyses are needed to precisely identify the *PIK3CA* effect.

### 4.7. Relationship between colorectal cancer and *EIF2S3* gene expression

EIF2 complex is required for protein synthesis [20]. In our study, *EIF2S3* mRNA levels increased in both colorectal tumor tissues and peripheral blood samples compared to the control group. This increase is only important for the change in the level of *EIF2S3* mRNA expressed in the peripheral blood of the cases. This increase is especially important in stages I and III peripheral blood samples. There are not many studies in the literature for *EIF2S3* mRNA analysis. According to the data obtained in our study, the increase in *EIF2S3* mRNA level in peripheral blood samples stands out in colorectal cancer cases and our data contributes to these limited studies. Further study is therefore required to understand *EIF2S3* mRNA changes in peripheral blood samples of colorectal cancer patients.

### 4.8. Relationship between colorectal cancer and *EIF4A1* gene expression

To the best of our knowledge, there are no more studies in the literature regarding *EIF4A1* mRNA gene expression changes. In our study, *EIF4A1* mRNA level in colorectal tumor tissues decreased compared to the control group, while it increased in peripheral blood compared to the control group. This increase is important both in the expression in general peripheral blood and especially in peripheral blood stages I and III. The malignant phenotype is the result of largely irregular gene expression. Transformed cells are due to not only a global increase in protein synthesis, but also a situation where pro-oncogenic mRNAs increase translationally. Such mRNAs have been shown to have longer and more structured 5p-UTRs that require high levels of eukaryotic initiation factor 4A (*EIF4A1*) helicase activity for effective transcription. Therefore, *EIF4A1* has begun to attract attention for cancer therapy. In order to be used as a biomarker in early diagnosis, detailed studies should be developed on the mechanisms that make specific mRNAs dependent on *EIF4A1* activity [60]. According to the results presented in this study, the increase in *EIF4A1* mRNA level in peripheral blood samples stands out in colorectal cancer cases and our results further contributes to these limited studies. Further study is therefore required to understand *EIF4A1* mRNA changes in peripheral blood samples of colorectal cancer patients.

## 5. Conclusion

The results reported in this study appears to suggest that the increase in *TRIP13* and *CPEB4* mRNA levels in peripheral blood samples of colorectal cancer cases may be a potential biomarker in early stage diagnosis of colorectal cancer. Considering the results related to *EIF2S3* and *EIF4A1* mRNA changes in the patients with colorectal cancer, the increase in mRNA levels in peripheral blood samples is remarkable. The major differences in mRNA levels in peripheral blood samples and tumor tissue samples likely reflect the tissue-specific specific regulatory mechanisms for related gene. Increases in the level of mRNA observed in the early stage of colorectal cancer suggest that relevant genes may play a role in carcinogenesis. Our data contains genetic information that may contribute to existing procedures in terms of diagnosis and prognosis in patients with colorectal cancer.

## Informed consent

This study was approved by the Ethics Committee of Afyonkarahisar Health Sciences University (2018/2 No: 39) and all patients provided informed consent.
